# GlassesValidator: A data quality tool for eye tracking glasses

**DOI:** 10.3758/s13428-023-02105-5

**Published:** 2023-06-08

**Authors:** Diederick C. Niehorster, Roy S. Hessels, Jeroen S. Benjamins, Marcus Nyström, Ignace T. C. Hooge

**Affiliations:** 1 Lund University Humanities Lab and Department of Psychology, Lund University, Lund, Sweden; 2 Experimental Psychology, Helmholtz Institute, Utrecht University, Utrecht, Netherlands; 3 Experimental Psychology, Helmholtz Institute & Social, Health and Organisational Psychology, Utrecht University, Utrecht, Netherlands; 4 Lund University Humanities Lab, Lund University, Lund, Sweden; 5 Experimental Psychology, Helmholtz Institute, Utrecht University, Utrecht, Netherlands

**Keywords:** Eye tracking, Data quality, Calibration, Validation, Accuracy, Reporting practices

## Abstract

According to the proposal for a minimum reporting guideline for an eye tracking study by Holmqvist et al. (2022), the accuracy (in degrees) of eye tracking data should be reported. Currently, there is no easy way to determine accuracy for wearable eye tracking recordings. To enable determining the accuracy quickly and easily, we have produced a simple validation procedure using a printable poster and accompanying Python software. We tested the poster and procedure with 61 participants using one wearable eye tracker. In addition, the software was tested with six different wearable eye trackers. We found that the validation procedure can be administered within a minute per participant and provides measures of accuracy and precision. Calculating the eye-tracking data quality measures can be done offline on a simple computer and requires no advanced computer skills.

## Introduction

Eye trackers enable recording eye movements and gaze behavior. According to the proposal for a minimum reporting guideline for an eye tracking study by Holmqvist et al. (2022), the data quality of the gaze position signal provided by the eye tracker should be reported. Data quality is often reported in terms of how closely the estimated gaze direction agrees with the actual gaze direction (accuracy), how repeatable the estimated gaze direction is (precision)^1^ and how many samples are invalid (data loss). Holmqvist et al. (2012, 2022) provide a good description of these data quality measures and how they can be operationalized (see also, e.g., Niehorster et al., 2020; Hooge et al., 2022). In this paper, we describe a no-frills method to determine accuracy and precision for data collected with a wearable eye tracker. The method involves a validation procedure to be used after any calibration of the eye tracker using the eye tracker’s standard methods or alternative methods such as Babcock and Pelz (2004); Evans et al. (2012); Santini et al. (2017). Wearable eye trackers are also referred to as head-mounted eye trackers (Elmadjian et al., 2018), mobile eye trackers (Schneider et al., 2018), and eye tracking glasses (Ye et al., 2012).

**Fig. 1:**
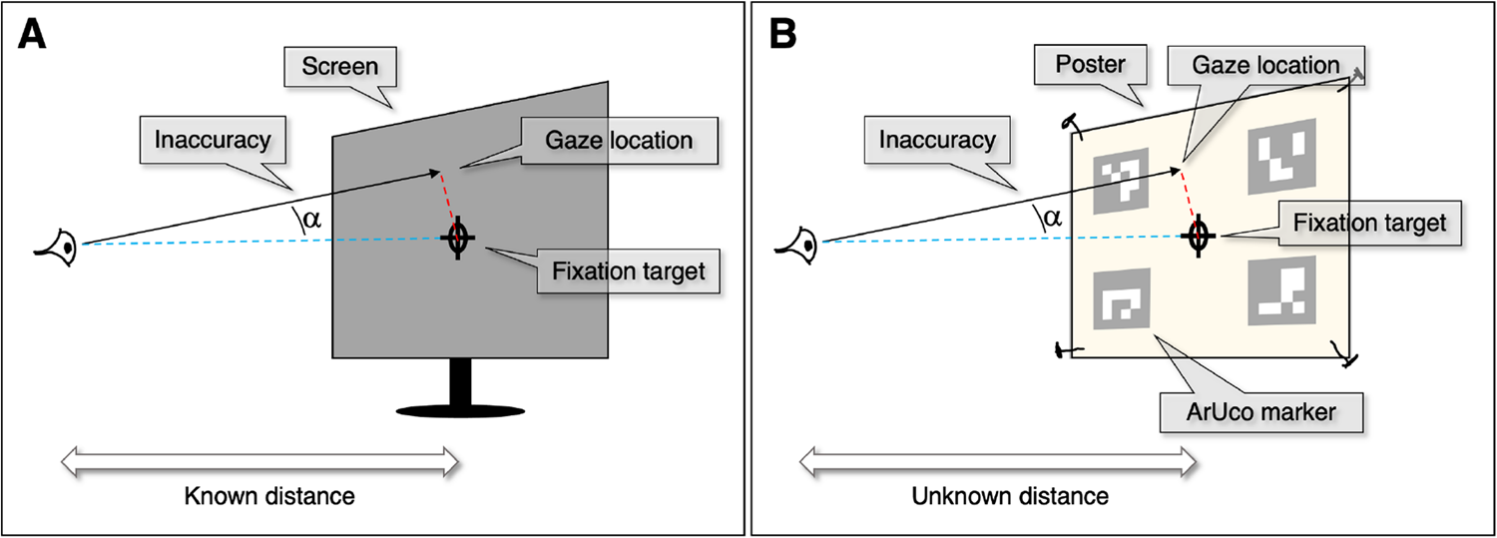
Calculating accuracy. **A** For a screen-based setup, typically both the physical distance between the gaze position and the fixation target and the viewing distance are known, making calculation of the (in)accuracy straightforward. **B** For a head-worn eye-tracker setup, typically the viewing distance and the physical gaze position are unknown. The use of ArUco markers of known spatial configuration and size enables estimating accuracy in degrees

How to determine the eye-tracking data quality for head-fixed (e.g., tower-mount) and head-boxed (e.g., remote, see Valtakari et al., 2021) eye trackers is well described in the literature (e.g., Holmqvist and Nyström et al., 2011; McConkie, 1981; Niehorster et al., 2020). Determining the data loss for wearable eye trackers is no different than determining it for head-fixed or head-boxed eye-tracking set ups. However, determining accuracy for wearable eye trackers is not straightforward because these eye trackers are usually used without a screen. We will illustrate this practical problem by means of (1) detailing how accuracy is determined for head-fixed or head-boxed eye tracking setups and by (2) comparing this with a realistic and representative example of how it is done for a wearable eye tracker.

The simplest method for estimating the (in-)accuracy of an eye tracker is to show a single fixation point on a computer screen (Fig. 1A) and instruct the participant to fixate this target. The angle ($\alpha$) between the line from the eye to the fixation target and the reported gaze direction is the error, the inaccuracy. This procedure can also be performed for other or multiple fixation targets and the resulting inaccuracies can also be averaged, depending on what is appropriate for the experiment. In the current example, we limit ourselves to determination of the inaccuracy using a single fixation target. To be able to determine the inaccuracy ($\alpha$), two distances must be known: (1) the distance from the eye to the screen and (2) the distance on the screen between the fixation target and the gaze position. The position of the eye in front the screen may be fixed by placing the participant in a chin- and forehead rest. If we would attempt to repeat this so-called validation procedure for an experiment with a wearable eye tracker, we immediately run into a problem: there is no computer screen in the experiment setup and thus no fixation target. The solution might appear obvious; use a computer screen and let the participant view a fixation point on this screen from a fixed distance. However, before we choose this seemingly simple solution, we will first sketch a realistic experimental scenario where wearable eye trackers are used in the field.

Consider the following scenario (inspired by studies such as Diaz et al., 2012; van Maarseveen et al., 2016; van Biemen et al., 2022). A researcher wants to investigate penalty shooting in soccer players. In order to do this, the researcher brings with them two pairs of eye tracking glasses (one for the goalie and one for the shooter). The penalty shooting takes place on Saturday morning on a training pitch. There are no power sockets on the training field and the lighting conditions vary; sometimes there is direct sunlight, sometimes the sun is hidden behind a cloud. Conducting such field research is further complicated because some of the football players are energetic guys with a short attention span; they do not have much patience and they make lame jokes all the time. The researcher needs all her attention to instruct the football players and the goalkeepers to make sure that they do what she wants. It would, in principle, be possible for the researcher to bring computers and computer screens with them, but it makes the operation of the researcher unnecessarily more difficult. Instead, she needs another way to easily and quickly determine data quality for their recordings.

In this article, we present glassesValidator, a method to easily and quickly determine the accuracy (and also the precision) of a wearable eye tracker in a field study. glassesValidator is a simple validation procedure that uses a poster with fixation targets and accompanying Python software to determine data quality measures. This software is deployed offline. The second part of this article consists of the evaluation of our new method.

## Tool description

The glassesValidator validation procedure consists of two parts, (1) a poster that is used during a recording, and (2) Python software for offline processing of the recording to estimate data quality measures. glassesValidator is available from https://github.com/dcnieho/glassesValidator as pip-installable Python code that support Windows, MacOS and Linux. The glassesValidator manual is found on the same website. Furthermore, standalone executables are provided for Windows. The glassesValidator package can be installed directly from Python using the command python -m pip install glassesValidator.

### The poster

**Fig. 2:**
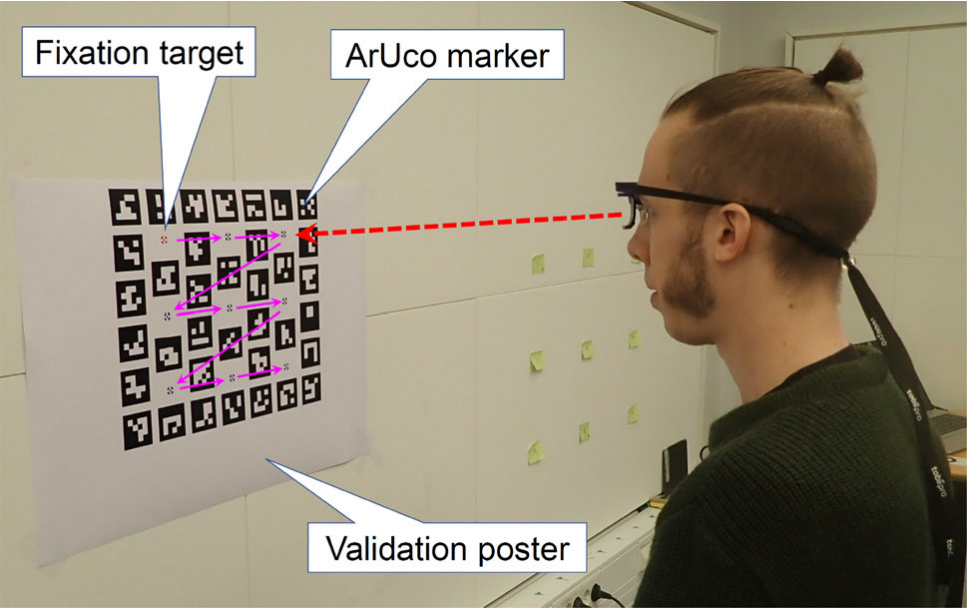
The second author viewing the default glassesValidator poster, which is hung such that the top row of fixation targets is approximately at eye height. The poster has nine fixation targets, the top left of which is red while the other eight are blue. The poster furthermore contains an array of 2D barcodes (ArUco markers) that enable determining gaze position on the poster and localization of the participant

As sketched in the Introduction, when performing a wearable eye-tracking study out in the field, the experimenter who wishes to determine the data quality of their recordings is faced with the problem of how to perform the validation procedure. That is, how do they display fixation targets at a known distance to their participants? Suitable fixation targets are often not readily available in the environment, and even if they were, the distance from which they are viewed would likely be unknown. We therefore designed a poster that solves both of these problems. The poster is shown in Fig. 2. The poster consists of two types of elements, (1) fixation targets and (2) specially designed 2-D barcodes called ArUco markers (Garrido-Jurado et al. , 2016). The fixation targets on the poster provide clear locations that the participant can be instructed to look at and provide a predefined looking order that the participant can easily be instructed to follow during the validation procedure. The fixation targets on the poster are placed such that they cover a $20 \times 17.5^{\circ }$ range at a perpendicular viewing distance of 60 cm (about an arm’s length). The array of ArUco markers of known spatial configuration and size serve as fiducial markers that enable a computer to automatically determine where on the poster a participant is looking according to the eye tracker, as well as their otherwise unknown viewing position and distance.

While glassesValidator comes with the default poster described above, it is fully customizable. The number, size and position of both fixation targets and fiducial markers can be varied freely, as long as all are placed on the same plane. Furthermore, in lieu of a poster, glassesValidator can be used with fixation targets and ArUco markers that are stuck to a wall, table or other flat surface, as long as their relative positions are known. Instructions for creating or modifying posters as well as using other target and marker spatial configurations are provided in the glassesValidator manual. Users should consider whether the range of viewing angles spanned by the gaze targets, as well as the distance from which they are viewed, is appropriate for their experiment and adapt the target and fiducial marker configuration if needed.

### The software

A wearable eye-tracking recording typically consists of a set of data files containing at least a list of gaze positions and a video taken with the eye tracker’s scene camera, showing what was in front of the participant during the recording. The gaze positions in the data file are positions on the scene video. The software part of the glassesValidator package enables estimating accuracy from such a wearable eye-tracking recording with minimal input from the experimenter. glassesValidator is currently able to import and process recordings made with the following eye trackers:

- Pupil Core
- Pupil Invisible
- SeeTrue
- SMI ETG 1 and ETG 2
- Tobii Pro Glasses 2
- Tobii Pro Glasses 3

How does glassesValidator derive the accuracy in degrees of a recording from the gaze data and the eye tracker’s scene video of a participant looking at the glassesValidator poster? As described above, two distances are needed to determine accuracy in degrees. Here we describe the required processing steps that deliver the two distances (the viewing distance between the eye and the poster, and the distance between the fixation target and the gaze point on the poster, cf. Fig. 1).

#### Viewing position

How does the researcher determine the position from which the participant viewed the poster? GlassesValidator offers two modes of operation: (1) The researcher can assume a fixed perpendicular viewing distance and provide this to the glassesValidator software. In this mode, it is assumed that the eye is located exactly in front of the center of the poster and that the poster is oriented perpendicularly to the line of sight from this assumed viewing position. (2) If the eye tracker’s scene camera is calibrated (i.e., its properties such as focal length and distortion parameters have been estimated by a calibration procedure), it is possible to use the array of ArUco markers to estimate the position and orientation of the participant relative to the poster at each time point during a validation. The position and orientation of the participant relative to the poster then allows estimating the viewing distance. This mode of operation is robust to participants that are at a different viewing distance and participants who do not maintain a single assumed viewing distance. If a calibration for the eye-tracker scene camera is not available, the experimenter can perform this calibration themselves and provide it for glassesValidator to use^2^ (see, e.g., Hooge et al., 2022).

#### Distance between fixation target and gaze point

What is required to determine the distance between the fixation target and the gaze point on the poster? First, gaze position must be transformed from the scene video reference frame to the plane of the poster containing the fixation targets. Similar to previous work (e.g., Santini et al., 2017; MacInnes et al., 2018; Niehorster et al., 2020; and the Pupil Labs surface tracker), the array of fiducial markers (ArUco markers in this case) of known spatial configuration and size allows performing this transformation.

Once the gaze data is transformed to gaze positions on the poster, one must decide which parts of the gaze data constitute fixation on each of the fixation targets (cf., e.g., Holmqvist, 2015; Niehorster et al., 2021). To associate gaze data with fixation targets, glassesValidator first uses the I2MC fixation classifier (Hessels et al., 2017) to classify the fixations in the gaze position data on the poster. Then, for each fixation target, the nearest fixation in poster-space that is at least 50 ms long is selected from all the classified fixations during the validation procedure. This matching is done in such a way that no fixation is matched to more than one fixation target. The mean of the gaze positions on the poster that comprise the selected fixation is then used as the gaze position associated with a fixation target.

Now that the gaze position on the poster and the viewing position of the participant have been determined, accuracy is straightforwardly computed as the angle between the vector from the participant to the gaze point and the vector from the participant to the fixation target (recall Fig. 1).

Similarly, information about the viewing position of the participant together with the gaze positions on the poster also enables calculating precision in degrees. Specifically, two often-used measures of precision, the RMS sample-to-sample distance (RMS-S2S) and the standard deviation (STD, see for both Niehorster et al., 2020), are calculated as follows. First, for all gaze positions during the fixation selected for a fixation target (see above), the angular offset of each gaze position to the fixation target is calculated. Then, standard formulae computing RMS-S2S and STD are applied.

## Evaluation

GlassesValidator has been evaluated for overall usability with data collected in a short experiment and with data collected by Hessels et al. (2022). Furthermore, data from these experiments were used to investigate two specific issues regarding the use of glassesValidator. These two experiments, investigating (1) the effect of instruction on accuracy, precision and gaze range, and (2) the error introduced in estimated accuracy when assuming a fixed viewing distance in the calculations, are presented next. The gaze point on the scene video is used for the analysis for both experiments, which is the default mode of operation of glassesValidator. How the gaze point is determined and whether it incorporates signals from one or both eyes may depend on the eye tracker.

### Effect of instruction on accuracy, precision, and range of gaze directions

Firstly, we investigated what are suitable instructions for the participant. When a participant stands in front of the poster and is instructed to fixate all nine fixation markers, there are two extreme ways in which they could carry out this instruction. First, the participant could keep their head still and move only their eyes to fixate the fixation markers. Second, the participant might make combined head-eye movements to the fixation markers with their head oriented such that each fixation marker is fixated in the middle of their oculomotor range. In this case, the participant would look to the fixation markers with their head oriented such that their nose points to each fixation marker. We do not know what is the natural way in which participants would execute the task of fixating the nine fixation markers. We performed an experiment as part of Hessels et al. (2022) to investigate how participants fixate the fixation targets.

**Fig. 3:**
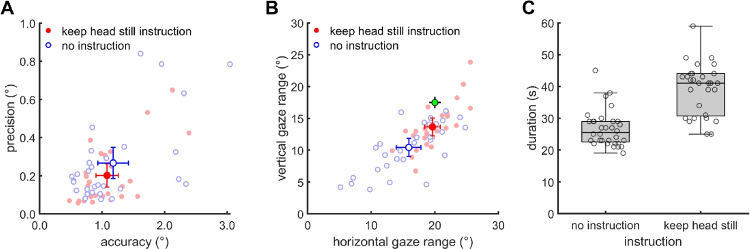
Analysis of validation procedure and gaze data quality. **A** Accuracy and precision: *solid red points* represent values for the “keep head still” instruction and *open blue points* represent no specific head movement instruction. *Pale-colored points* represent individual recordings. **B** Horizontal and vertical gaze range. The *green cross-hair dot* indicates the theoretical prediction for the gaze range with a completely still head ($20^{\circ }$ horizontally and $17.5^{\circ }$ vertically). **C** Boxplots showing the distribution of time it took to perform a validation. *Dots* indicate the duration of individual validations. The *error bars* in panels A and B indicate 95% confidence intervals

#### Methods

This investigation was performed as part of an experiment conducted during a science festival. A report on this experiment has previously been published, see Hessels et al. (2022). In total, the recordings of 61 participants were included in the analysis presented here (one further participant’s recording could not be used because the recording was inadvertently started only after the validation procedure was performed). Participant age range was 19–69 years, 40 were female and 21 male. Tobii Pro Glasses 2 were used for all recordings. About half of the participants ($n = 29$) were instructed to look at the nine fixation points using only their eyes while keeping their head still, while the other participants ($n = 32$) were only told to look at the fixation points. These instructions were provided by two operators; each was trained to provide one of the instructions. For each recording, the range of horizontal and vertical gaze directions was determined by subtracting the most negative (leftward or upward) from the most positive (rightward or downward) gaze direction observed during the validation procedure. Specifically, the mean gaze direction during fixations to the fixation markers as selected by glassesValidator was used for this analysis.

#### Results and discussion

Four recordings were excluded from the data quality and gaze range analyses presented here because data loss in these recordings was too high (16–31$\%$).

Do the instructions lead to differences in estimated data quality? To examine this, in Fig. 3A we plot the estimated accuracy of each participant against the estimated RMS-S2S precision. Data for participants who were instructed to keep their head still are represented by solid red points, whereas data for participants who received no such instruction are blue open points. No systematic differences in either accuracy or precision were observed as indicated by the overlap in the 95% confidence intervals associated with the mean accuracy and precision of the two instruction conditions.

Do the instructions affect the gaze range exhibited by participants? Figure 3B shows a scatterplot of the horizontal and vertical gaze ranges. There was significant variability in the range covered by participants’ gaze behavior regardless of whether participants were instructed to keep their head still or not. Nonetheless, both the vertical and horizontal gaze ranges were larger when participants were instructed to keep their head still than when they were not as indicated by the non-overlapping 95% confidence intervals. Furthermore, the horizontal gaze range displayed by participants who were instructed to keep their head still was not different from the range expected with a completely still head ($20^{\circ }$), as indicated by the confidence interval overlapping the green dot indicating the expected range. The vertical gaze range was, however, less than expected ($17.5^{\circ }$) regardless of instruction.

Last, to evaluate whether the glassesValidator procedure is quick and easy to use, we examined how much time it took to perform a validation. This time was coded by the first author based on the scene video and audio from each recording and includes both the time it took to instruct participants and the time it took for participants to perform the validation procedure. The resulting validation durations are displayed in Fig. 3C. Although providing the instruction to keep the head still appears to have taken extra time, this difference might not be due to the different instructions but could be because the two instructions were delivered by two different operators. Importantly, nonetheless, performing a validation was fast for all participants regardless of instruction (less than 1 min).

**Fig. 4:**
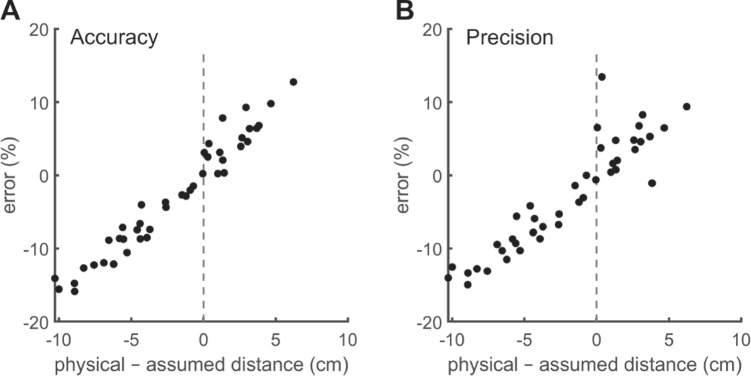
Error in accuracy **A** and precision **B** estimation when assuming a perpendicular viewing distance of 60 cm. Each *dot* is the average of the nine points of a validation procedure, of which there were two per eye tracker per participant. The *dotted line* indicates the assumed viewing distance (60 cm)

In summary, we found that accuracy and precision estimated with our method did not differ between recordings of participants who were instructed to keep their head still and participants who received no such instruction. The instruction did however affect gaze behavior: the range of gaze angles shown by participants who were instructed to keep their head still was larger than that of participant who received no such instruction. We think that the gaze behavior we observed when instructing participants to keep their head still during the validation procedure is more representative of the range of gaze angles that might be observed in experimental research. We therefore recommend including instructions to participants to keep their head still and move only their eyes when looking at the fixation targets on the glassesValidator poster.

### Assuming a fixed viewing position

As described in the “Viewing position” section above, glassesValidator has two modes of operation for determining accuracy. Either a fixed viewing position and perpendicular viewing distance are assumed for all calculations, or, if possible, the position and orientation of the participant relative to the poster is continuously estimated and used instead. When the viewing distance, viewing position or poster orientation used for calculating accuracy is incorrect, this would introduce an error in the estimated accuracy. For instance, the accuracy values would be either too large or too small depending on whether the assumed viewing distance was smaller or larger than the physical viewing distance. To evaluate what error is introduced by assuming a fixed viewing position, we calculated the error in accuracy and precision as function of the physical distance.

#### Methods

This investigation was performed during a brief experiment that was performed at the same time as the wearable eye tracking test of Hooge et al. (2022). Gaze of four male participants (age 31–56 years) was recorded using six different eye trackers while they performed the validation procedure both before and after the recordings of Hooge et al. (2022). The six eye trackers were a Pupil Core, a Pupil Invisible, a SeeTrue, an SMI ETG 2 60-Hz, a Tobii Pro Glasses 2, and a Tobii Pro Glasses 3. To determine the error in accuracy and precision as function of the physical viewing distance, accuracy and precision were estimated for the validation episodes in the resulting recordings using the two different operation modes of glassesValidator. Accuracy and precision were calculated using 1) an assumed fixed viewing position and perpendicular viewing distance of 60 cm; or 2) a continuously updated viewing position of the participant. The error was then determined as $accuracy\_fixed\_dist/accuracy\_physical\_dist * 100\%$, and similarly for precision. Hooge et al. (2022) performed their own calibration of the SeeTrue’s scene camera. This calibration was used for the evaluation presented here.

#### Results and discussion

Figure 4 shows the error in computed accuracy (panel A) and precision (panel B) when assuming a perpendicular viewing distance of 60 cm, as a function of the physical viewing distance. The error in accuracy and precision increases proportionally to the difference between the assumed and physical viewing distance. The percentual errors shown in the figure corresponded to $0.001-0.55^{\circ }$ for accuracy and $0-0.20^{\circ }$ for precision. Are these errors sufficiently large to always recommend using a camera calibration when determining data quality? Here we do not provide the reader with thresholds for acceptable levels of error and thus do not answer that question. As McConkie (1981) points out: “it is not appropriate to adopt standards concerning what is acceptable data; that varies with the nature of the questions being studied”. As such, the reader should determine for themselves if the error involved is acceptable for their use case.

**Fig. 5:**
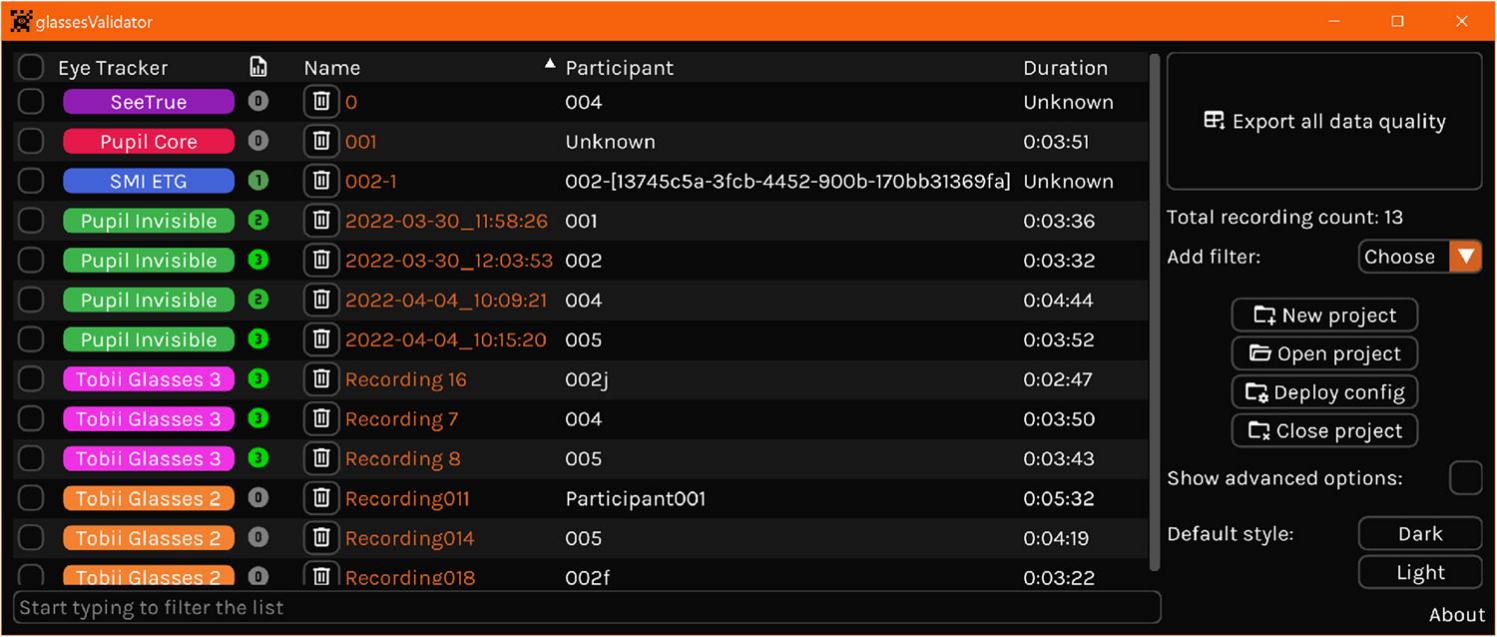
A screenshot of the glassesValidator graphical user interface showing a listing of several imported recordings from different eye trackers. The state of each recording is indicated: 0: not imported; 1: imported into the glassesValidator project; 2: episodes containing a validation have been indicated for the recording; 3: data quality has been determined. A summary of data quality measures can be exported with the button in the top right. Also shown is the functionality for managing glassesValidator projects, including the “deploy config” button that can be used to change the default glassesValidator configuration, such as the poster layout

## Workflow

A suggested user workflow for using the poster and processing a recording to estimate accuracy is detailed in this section.

To easily process recordings without needing to know programming, the glassesValidator package includes a graphical user interface (GUI, Fig. 5) that can be used to perform all processing. Below we describe an example workflow using the GUI. Advanced users can however opt to call all the GUI’s functionality directly from their own Python scripts without making use of the graphical user interface. The interested reader is referred to the glassesValidator manual for further details regarding how to use the glassesValidator functionality directly from their own scripts.

1. Before recording, the researcher prints the poster included with glassesValidator on A2 paper. In order to check that the poster was printed at the correct scale, one should measure the sides of the ArUco markers. We strongly recommend performing this check because printers may not be calibrated. In the case of the default glassesValidator poster, the distance between the left side of the left-most column of ArUco markers and the right side of the right-most column of ArUco markers should be 35.6 cm (each ArUco marker should have sides that are 4.19 cm long). If the poster was printed at the wrong scale, one must adapt the glassesValidator configuration to match the size and position of the ArUco markers and fixation targets on your poster.
2. Before recording, the researcher hangs the printed poster on a flat surface, such as a wall. Vertical positioning of the poster depends on the experiment setting, but we think that hanging the poster such that the top row of fixation targets is at eye height for an average-length participant is a suitable default.
3. The operator positions the participant in front of the glassesValidator poster. An easy method for positioning the participant is to ask them to stretch their arm out straight forward and stand at a distance where their fist touches the poster. The operator then issues the following suggested instructions: “look at the nine fixation targets in reading order for one second each. Start with the top-left (red) target. When looking at the fixation targets, keep your head as still as possible, move only your eyes.” These verbal instructions could be accompanied by pointing at the fixation targets in the desired looking order to further help the participant to follow the instructions.
4. To start calculating accuracy and precision, the researcher imports the recordings for which data quality should be determined into a glassesValidator project, for instance by drag-dropping a folder with recordings onto the glassesValidator GUI window and selecting the import action.
5. Once imported, the researcher indicates which episode(s) of each recording contain a validation using a graphical interface included with glassesValidator.
6. The recordings are then further processed automatically, and data quality is determined for validation episodes in the recording.
7. Finally, once all recordings have been processed, the researcher exports the data quality measures from the recordings in the project into a summary Excel file. This summary function can optionally average the data quality values over the fixation targets for each recording.

## Applications

The accuracy and precision calculated for wearable eye tracking recordings can be used for at least the following applications:

1. Reporting data quality in publications. According to (Holmqvist et al., 2022), the data quality of the gaze position signal provided by the eye tracker should be reported for all eye tracking studies. glassesValidator provides these values.
2. Evaluating whether data quality deteriorates during the course of a recording. Data quality during a recording may deteriorate for instance because the wearable eye tracker shifted on the participant’s head or due to participant behavior (cf. Niehorster et al., 2020; Hooge et al., 2022). To evaluate data quality during the recording, the operator may for instance conduct an additional validation at the end of the recording or insert validations during the recording. The glassesValidator software can handle recordings containing multiple validations, allowing the researcher to check whether data quality is sufficient for their study throughout the recording.
3. Exclusion of recordings with insufficient data quality. Excluding a number of recordings from further analysis is a common occurrence in eye tracking studies. However, quantitative values representing data quality are needed to enable the researcher to select only those recordings with sufficient data quality for inclusion in their study. glassesValidator provides researchers with the values they need to make this selection.

## Data Availability

The glassesValidator tool is available at https://github.com/dcnieho/glassesValidator. The experiments were not preregistered.

## Footnotes

1 Or more precisely, precision is “the closeness of a set of repeated gaze position measurements obtained under identical conditions (i.e., obtained from an eye that has not rotated)” (Niehorster et al., 2020, p. 4).

2 At the time of writing, five of the six wearable eye trackers supported by glassesValidator provide the calibration of their scene camera, and glassesValidator will by default use this calibration for these eye trackers. Currently, only the SeeTrue does not provide a camera calibration, and glassesValidator therefore by default uses an assumed fixed viewing distance for this eye tracker.
